# Dysregulation of Histone Deacetylases Inhibits Trophoblast Growth during Early Placental Development Partially through TFEB-Dependent Autophagy-Lysosomal Pathway

**DOI:** 10.3390/ijms241511899

**Published:** 2023-07-25

**Authors:** Peixin Wang, Chenqiong Zhao, Hanjing Zhou, Xiaona Huang, Hanqi Ying, Songying Zhang, Yibin Pan, Haiyan Zhu

**Affiliations:** 1 Assisted Reproduction Unit, Department of Obstetrics and Gynecology, Sir Run Run Shaw Hospital, School of Medicine, Zhejiang University, Hangzhou 310016, China; 2 Key Laboratory of Reproductive Dysfunction Management of Zhejiang Province, Hangzhou 310016, China

**Keywords:** recurrent spontaneous abortion, autophagy, epigenetic modification, acetylation, histone deacetylase inhibitor (HDACI), transcription factor EB, nuclear translocation, pathological course

## Abstract

Dysregulated biological behaviors of trophoblast cells can result in recurrent spontaneous abortion (RSA)—whose underlying etiology still remains insufficient. Autophagy, a conserved intracellular physiological process, is precisely monitored throughout whole pregnancy. Although the exact mechanism or role remains elusive, epigenetic modification has emerged as an important process. Herein, we found that a proportion of RSA patients exhibited higher levels of autophagy in villus tissues compared to controls, accompanied with impaired histone deacetylase (HDAC) expression. The purpose of this study is to explore the connection between HDACs and autophagy in the pathological course of RSA. Mechanistically, using human trophoblast cell models, treatment with HDAC inhibitor (HDACI)-trichostatin A (TSA) can induce autophagy by promoting nuclear translocation and transcriptional activity of the central autophagic regulator transcription factor EB (TFEB). Specifically, overactivated autophagy is involved in the TSA-driven growth inhibition of trophoblast, which can be partially reversed by the autophagy inhibitor chloroquine (CQ) or RNA interference of TFEB. In summary, our results reveal that abnormal acetylation and autophagy levels during early gestation may be associated with RSA and suggest the potential novel molecular target TFEB for RSA treatment.

## 1. Introduction

Recurrent spontaneous abortion (RSA), characterized by three or more consecutive spontaneous abortions before 20 weeks of gestation, has become one of the trickiest clinical challenges in the field of reproduction, accounting for 10–15% of all pregnancies and affecting 1–5% of fertile women worldwide [1]. The etiology reported involves multiple factors but cannot be defined exactly because of its flexibility. As specialized cells in the placenta, trophoblasts mediate central functions in each period of pregnancy and control the pregnancy outcomes [2,3]. Cytotrophoblast (CTB) progenitors can give rise to cells (EVTs) that invade the uterus and vasculature or generate multinuclear syncytiotrophoblasts (STBs) that form on the surface of the chorionic villi during the first trimester of human gestation [4,5]. Notably, human trophoblast stem (TS) cells, which were successfully derived from human blastocysts (TS^blast^) or cytotrophoblast (TS^CT^) cells in 2018, have been proved to be meritorious in vitro models for the characteristic analysis of human trophoblast cells based on their similar transcriptome to the primary trophoblasts [3,6].

Autophagy is an evolutionarily orchestrated metabolic process that depends on lysosomes to degrade and on the recycling of intracellular components to maintain homeostasis inside all eukaryotic cells [7,8,9]. A lot of autophagy-related genes (Atgs) and proteins were reported to be involved in the autophagic process. The conversion of LC3I to LC3II served as a marker of autophagy activation, while the degradation of p62 is considered to be an indicator of autophagic protein degradation [10]. Growing evidence has confirmed that autophagy can be induced under physiological or pathological conditions, which is closely associated with specific diseases, although the specific relationship is context-dependent [9,11,12]. In terms of the reproductive system, autophagy is also physiologically involved in early normal gestation, playing an important role in embryogenesis and implantation [8]. Dysregulation of autophagy has been reported in both preeclampsia and fetal growth restriction despite controversial conclusions, while few studies focused on its role in RSA [8]. However, the regulation of autophagy is complex, since this process is always guided by a series of differential genes expression, including transcription factors, signaling molecules and growth factors. Most studies have considered phosphoinositide 3 kinase (PI3K)/protein kinase B (AKT)/rapamycin (mTOR) and activation of the ERK1/2 as classic autophagy pathways, but paid little attention to the importance of epigenetics mechanisms [13]. As one of the crucial events of autophagy execution, the transcriptional program has emerged as a crucial regulatory mechanism, which may be of great significance to pregnancy outcome anticipation [12].

In recent years, the potential significance of epigenetics to the trophoblast of early gestation has been gradually recognized, which refers to the heritable regulation in gene expression caused by the posttranslational modification of protein complexes without alteration in the DNA sequence [14,15]. Histone acetylation, as one of the major modes, is a reversible process that is tightly controlled by a balance between histone acetyltransferases (HATs) and histone deacetylases (HDACs) [16]. This dynamic equilibrium has become an important determinant in cell metabolism based on its critical role as a regulator of chromatin accessibility and gene transcription [17]. Trophoblast epigenome is dramatically reprogramed during early gestation, whose failures are associated with placental diseases [18]. Similar to tumors, studies have confirmed that type I (HDAC1, 2, 3, and 8) and type II (HDAC4, 5, 7 and 9) of HDACs demonstrate high expression in the placenta, which indicates their certain roles in maintaining normal pregnancy [19,20]. HDAC1-null mice will die prenatally with a severe proliferation defect and general growth retardation [21]. Dysregulation of HDAC2 and HDAC9 is related to the pathogenesis of preeclampsia, which can repress the migration of trophoblast cells and regulate the cell-signaling pathways by modifying crucial molecules [19,22]. In addition, it has been reported that the impaired mitochondrial function and proinflammatory cytokine secretion in gestational diabetes mellitus are associated with decreased HDAC2 activity [23]. Histone deacetylase inhibitors (HDACIs) are a class of compounds that disrupt the balance of histone acetylation via interfering with the function of histone deacetylases, which are widely studied in the field of cancer. However, the effects of deacetylase inhibitors extend far beyond histones; other mechanisms including a large number of nonhistone targets such as heat shock proteins, cytoskeletal proteins and transcription factors, are involved in HDAC-mediated gene regulation [16,24]. In a variety of tumors, key mechanisms of autophagy have been proved to undergo extensive acetylation modification, which is associated with long-term transcriptional regulation [9]. However, no studies have explored the interaction of acetylation and autophagy in trophoblast cells [16,25,26,27]. Our clinical samples data revealed that impaired HDACs expression accompanied by exaggerated autophagy presented in the villi of RSA patients. Based on the similarity of biological behaviors between the tumor and trophoblast [28,29], we hypothesized that there may be a connection between aberrant activated autophagy and dysregulated HDACs during early pregnancy.

The transcription Factor EB (TFEB) is a master regulator of autophagic function and lysosome biogenesis, whose transcriptional activity is determined by the intracellular localization [30,31]. Nuclear TFEB can upregulate the expression of nearly two-thirds of autophagy-lysosome-related genes, including the lysosomal-associated membrane protein 1 (LAMP1) [32]. Interestingly, as a non-histone, the nucleoplasmic translocation of TFEB is also a favored target for various posttranslational modifications although the specific mechanism is still poorly understood. MTOR-mediated phosphorylation and other protein kinases, including GSK3β, Akt and PERK, have been demonstrated to influence the nuclear localization of TFEB [30,31,32,33]. Recently, more and more attention has been paid to the regulatory mechanism of acetylation. Acetylated TFEB was proved to have a much higher capability of nuclear translocation and DNA binding [34]. Notably, none of studies focused on the relationship between TFEB and acetylation in the trophoblast during early gestation, and whether TFEB is one of the targets in hyperacetylation-induced excessive autophagy under abnormal embryonic development remains unknown.

In the present study, we investigated the role of abnormal acetylation levels on placental autophagy, finding that hyperacetylation will induce autophagy but inhibit growth in the trophoblast, which may be related to pregnancy complication RSA. Our results further revealed the transcriptional mechanism of HDACI-induced autophagy. Intracellular hyperacetylation induced by HDACI can not only directly target autophagy related proteins, but also increase the transcriptional activity of non-histone TFEB and promote its nuclear translocation, contributing to the overactivated autophagy. Moreover, induced autophagy will further aggravate the growth inhibition of the trophoblast under the acetylated condition; the role of TFEB in this process was validated. Thus, we suggest that hyperacetylation and excessive autophagy in villi during the first trimester may be one of the predictors for pregnancy outcome, and TFEB may become a potential target for RSA therapy.

## 2. Result

### 2.1. Decreased HDAC Expression and Excessive Autophagy Levels in the Villi of RSA Patients during Early Gestation

**Figure 1:**
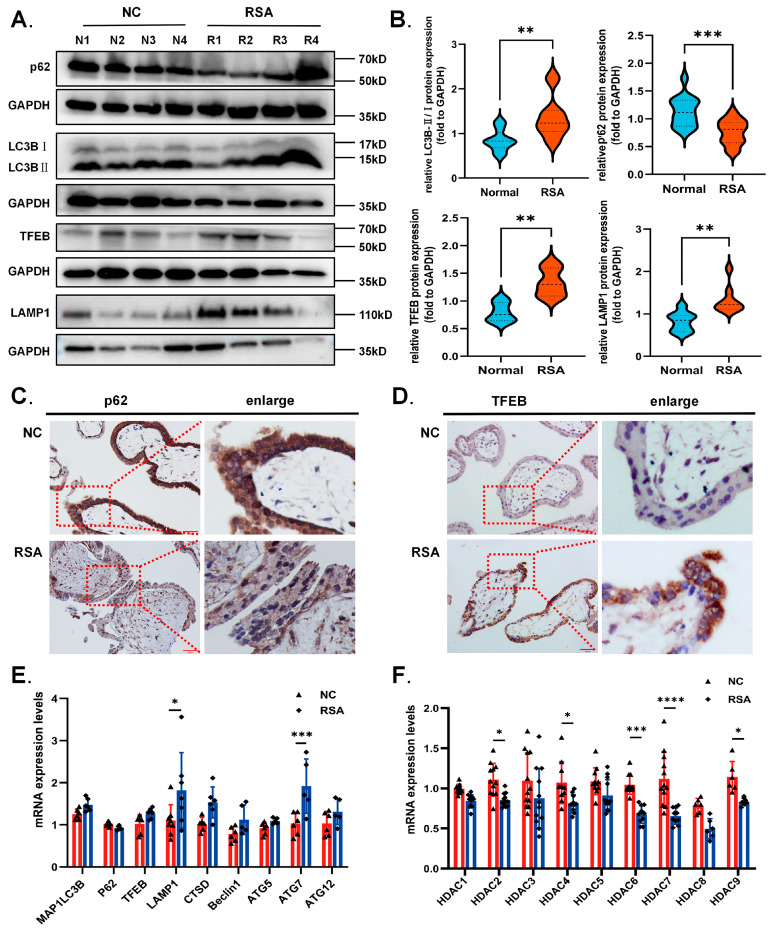
**The villus tissues of RSA patients exhibited higher autophagy levels and lower HDACs expression.** (**A**) Human first trimester villus samples from normal (*n* = 20) and RSA (*n* = 15) patients were used for Western blot. (**B**) Quantitative analysis of autophagy associated proteins LC3BII/I, p62, TFEB and LAMP1 in two groups. They were all normalized to GAPDH. (**C**,**D**) Representative images of p62 and TFEB IHC staining in normal and RSA villus tissue. Scale bar, 50 μm. (**E**,**F**) Relative mRNA expression of deacetylase HDAC1-7 and autophagy related genes in two groups were determined using RT-qPCR. Statistical analyses were performed by Student’s *t*-test and presented as the mean ± SD. (* *p* < 0.05, ** *p* < 0.01, *** *p* < 0.001, **** *p* < 0.0001). All experiments were performed in triplicate (*n* = 3).

To investigate whether the dysregulated autophagy level during early pregnancy is associated with RSA, we collected villus samples from placentas of patients with RSA (*n* = 15) and healthy controls (*n* = 20) ranging from the sixth to eighth week of gestation. The relevant experimental results are shown in Figure 1. We found that the expression of the autophagy signature protein p62 was significantly lower in RSA villi while the LC3B-II/LC3B-I ratio was higher compared to the control. Notably, the master transcriptional regulator of autophagy and lysosomal biogenesis TFEB accompanied with its downstream protein lysosomal associated membrane protein 1 (LAMP1) were also overexpressed in RSA patients (Figure 1A,B), suggesting that excessive autophagy and related lysosomal biogenesis may be involved in the occurrence of RSA. To further verify, we performed immunohistochemistry. Results demonstrated pale homogeneous staining for p62 but distinct staining for TFEB in the trophoblast layer of RSA villi, in line with the Western blot analysis (Figure 1C,D). In addition, these data were then confirmed by discrepancies in mRNA levels of autophagy-related-genes between the two groups (Figure 1E). Interestingly, excessive autophagy was coincided with abnormal HDACs expression, which were down-regulated in the RSA group as indicated by relative mRNA levels in villi (Figure 1F). Since the HDACs determine the acetylation level, we also detected the marker proteins of histone acetylation in villi. The results showed that the expression of acH3 and acH2B in RSA villi was higher than that in the normal control (Supplementary Figure S1). Thus, we further explored the relationship between decreased HDAC expression and overactivated autophagy under pathological conditions during early pregnancy based on the facts above.

### 2.2. HDAC Inhibitor TSA Induces Acetylation and Autophagy in Human Trophoblast Cells

**Figure 2:**
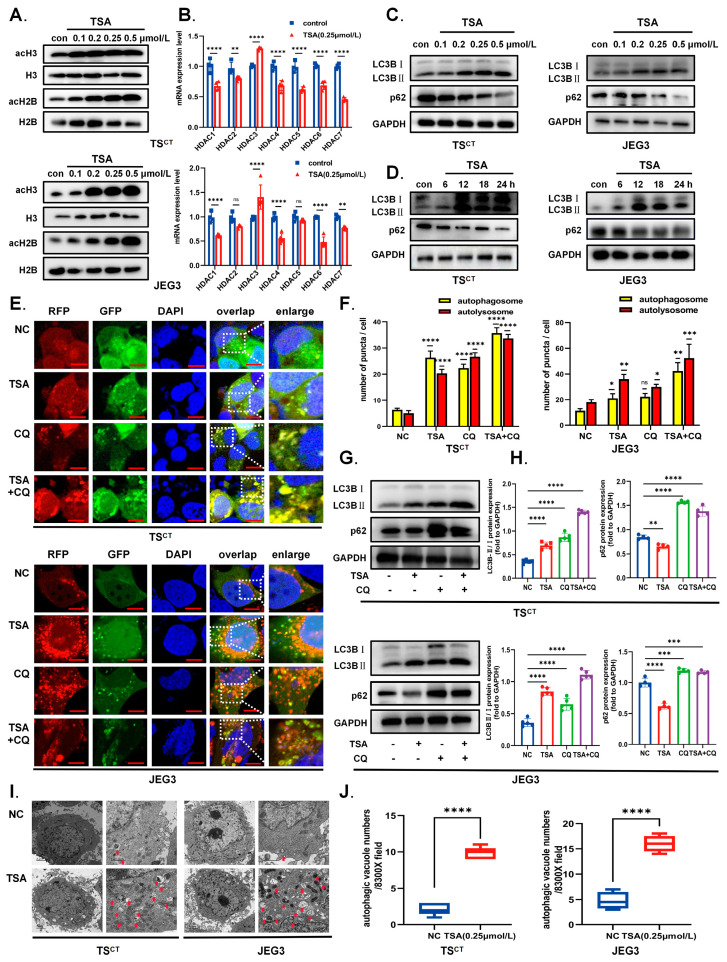
**TSA induced autophagy in trophoblast cells by decreasing HDACs expression.** (**A**) Human TS^CT^ cells and JEG3 cells were treated with different dose of TSA (0.1, 0.2, 0.25, and 0.5 μmol/L) for 12 h as designated. Cells were harvested and lysed for Western blot to detect the expression of acH3 and acH2B. GAPDH was used as internal control. (**B**) Relative mRNA expression of HDAC1-7 was estimated after TSA treatment (0.25 μmol/L, 12 h) using RT-qPCR. (**C**) Western blot revealing LC3BII/LC3BI ratio and p62 protein expressions in Human TS^CT^ cells and JEG3 cells varied with the concentration gradient of TSA (0.1, 0.2, 0.25, and 0.5 μmol/L) after 12 h treatment. (**D**) Western blot revealing LC3BII/LC3BI ratio and p62 protein expression in two types of cells by treatment with TSA (0.25 μmol/L) for the time indicated. (**E**) Representative images showing GFP^+^mCherry^+^puncta and GFP^-^mCherry^+^puncta after being infected with Ad-mCherry-GFP-LC3B adenovirus and treated with drugs respectively (TSA 0.25 μmol/L for 12 h; CQ 20 μmol/L for last 6 h of TSA treatment), Scale bars of TS^CT^: 20 µm; Scale bars of JEG3: 10 µm. (**F**) Quantitative analysis of GFP^+^mCherry^+^ yellow neutral puncta (autophagosomes) and GFP^-^ mCherry^+^ red acidified puncta (autolysosomes) per cell by ImageJ. (**G**) Human TS^CT^ cells and JEG3 cells were treated with the TSA (0.25 μmol/L) for 12 h, followed by treatment with or without CQ (20 μmol/L) for last 6 h before being collected for Western blot analysis. (**H**) LC3BII/LC3BI ratio and p62 levels were quantified by densitometric analysis, and normalized to GAPDH. (**I**) TEM images of Human TS^CT^ cells and JEG3 cells with or without TSA (0.25 μmol/L,12 h) including enlargements(right-side). Red arrows indicate autophagic vacuoles. (**J**) Quantification of autophagic vacuoles per 8300X field in each group of three independent experiments. Statistical analyses were performed by Student’s *t*-test and One-way ANOVA; The data were presented as the mean ± SD. (* *p* < 0.05, ** *p* < 0.01, *** *p* < 0.001, **** *p* < 0.0001 and ns means non-significant). All experiments were performed in triplicate (*n* = 3).

Trichostatin A, a broad-spectrum HDAC inhibitor, was used in the Human TS^CT^ cell model/Human-derived trophoblast JEG3 cell line. As shown in Figure 2A,B, the treatment of the trophoblast with TSA successfully down-regulated the expression of HDAC1-7 and increased the histone acetylation level (acH2B and acH3) compared with the control group. At the same time, autophagy was induced in both trophoblast cells in a time/dose-dependent manner, which can be indicated by the increase in LC3BII/LC3BI ratio (autophagosome marker) and the decrease in p62 protein (autophagy substrate) (Figure 2C,D). Additionally, as the LC3B-II accumulation can be induced by both autophagosome synthesis or undesirable degradation, we evaluated the LC3BII/LC3BI ratio and p62 levels in the absence and presence of CQ, which can inhibit late-stage autophagy. The relative accumulation of LC3B-II was accelerated and the degradation of p62 was blocked when CQ appeared in TSA group, further confirming the autophagy induction by TSA (Figure 2G,H). To analyze more visually, we transfected Ad-mCherry-GFP-LC3B adenovirus into trophoblast cells: red vesicles (GFP^-^mCherry^+^puncta) represent autolysosomes while yellow vesicles (GFP^+^mCherry^+^puncta) represent autophagosomes. The results of immunofluorescence under the confocal microscope indicated that TSA treatment increased the number of both the autophagosome and autolysosome, which is consistent with the Western blot analysis (Figure 2E,F). Moreover, the number of autophagic vacuoles was significantly increased in the TSA group, as determined by the transmission electron microscopy (Figure 2I,J). These data indicate that the HDAC inhibitor TSA positively modulated autophagy initiation via enhancing the acetylation level in trophoblast cells during early pregnancy.

### 2.3. HDAC Inhibitor TSA Promotes Nuclear Translocation and Transcriptional Activity of TFEB in Human Trophoblast Cells

**Figure 3:**
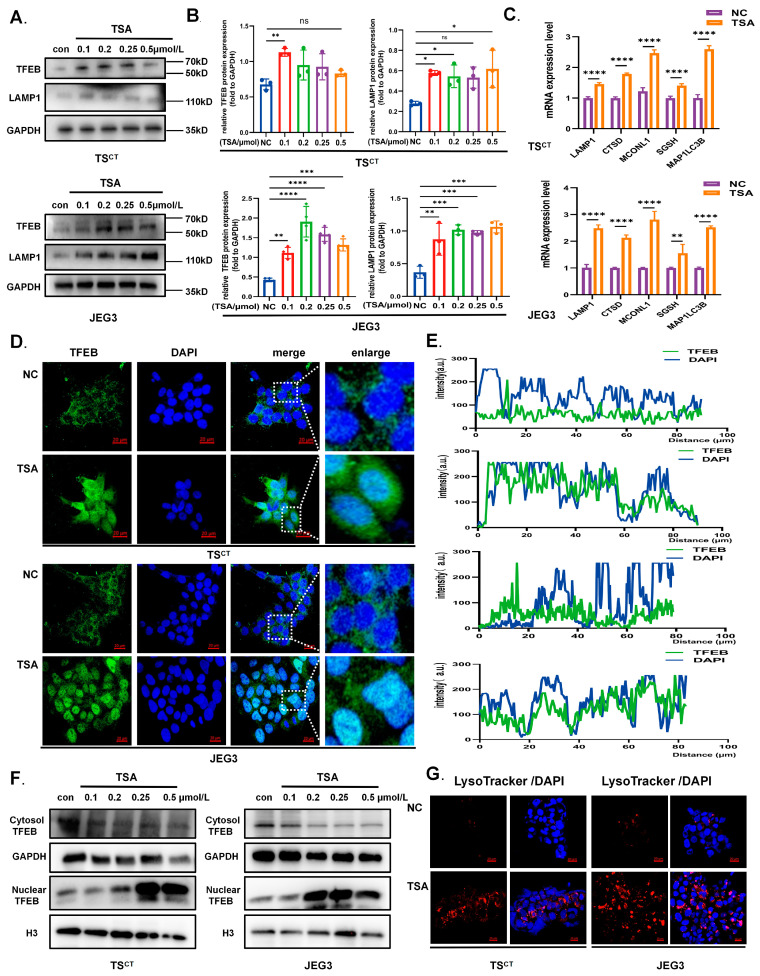
**TSA stimulated nuclear translocation of TFEB to increase transcriptional activity in trophoblast cells.** (**A**,**B**) Western blot revealing TFEB and LAMP1 protein expression in Human TS^CT^ cells and JEG3 cells by treatment with TSA (0.25 μmol/L) for 12 h and followed by quantitative analysis. (**C**) RT-qPCR analysis of TFEB transcriptional activity via detecting downstream target genes of TFEB including LAMP1,CTSD, MCONL1, SGSH, MAP1LC3B in TS^CT^ cells and JEG3 cells with or without TSA treatment. (**D**) Immunofluorescence detection about the effects of TSA (0.25 μmol/L, 12 h) on TFEB nucleus translocation in trophoblast cells. Scale bars = 20 μm. (**E**) Fluorescence quantitative analysis of TFEB and nucleus to determine cellular sublocalization of TFEB. (**F**) Western blot analysis of TFEB in the cytosol and nucleus of trophoblast cells after TSA treatment (0.25 μmol/L, 12 h). GAPDH and Histone H3 were used as quality controls for the cytosolic and nuclear fractions respectively. (**G**) LysoTracker staining in Human TS^CT^ cells and JEG3 cells treated with or without TSA (0.25 μmol/L, 12 h). Scale bars = 20 μm. Statistical analyses were performed by Student’s *t*-test and One-way ANOVA; The data were presented as the mean ± SD. (* *p* < 0.05, ** *p* < 0.01, *** *p* < 0.001, **** *p* < 0.0001 and ns means non-significant). All experiments were performed in triplicate (*n* = 3).

The transcription factor EB (TFEB) is involved in lysosomal biogenesis and molecular clearance simultaneously [35]. The subcellular localization of TFEB is essential for its transcriptional activity, which may become a transcriptional program target [35,36,37,38]. Interestingly, in our study, we found that intracellular hyperacetylation induced by TSA can not only directly target autophagy related proteins, but also increase the TFEB expression and contribute to its nuclear translocation, regulating autophagy at the transcriptional level. In addition, we observed that the lysosomal-associated membrane protein 1 (LAMP1), the membrane marker of lysosomes and downstream target gene of TFEB, increased in a dose-dependent manner after the TSA treatment in Human TS^CT^ cells and the JEG3 cell line, which is consistent with clinical results (Figure 3A,B). Furthermore, the immunofluorescence also revealed visually that the nuclear localization of TFEB increased after treatment with 0.25 μmol/L TSA. TFEB mainly localized in the cytosol normally while TSA significantly promoted TFEB nuclear translocation and increased the nuclear TFEB (Figure 3D,E). Western blot analysis also indicated that the expression of nuclear TFEB increased while cytosol TFEB decreased in a dose-dependent manner after TSA treatment (Figure 3F). As a transcription factor, TFEB can promote the expression of autophagic and lysosomal genes. Therefore, we examined the mRNA levels of TFEB target genes to further detect the transcriptional activity of TFEB. After TSA treatment, the expression of lysosomal and autophagic genes elevated significantly (Figure 3C). Moreover, TSA enhanced the staining of cells by LysoTracker Red, as measured by the confocal microscope (Figure 3G). These results suggest that TSA enhanced the transcriptional activity of TFEB by regulating nuclear translocation and promoting TFEB-dependent lysosome biogenesis. Furthermore, we knocked down nuclear HDACs (HDAC1/2/3/8) and cytosol-localized HDACs (HDAC4/5/6) in Human TS^CT^ cells using specific siRNAs and found that the knockdown of HDAC1/2/5/6 can also promote nuclear translocation to different degrees (Supplementary Figure S2). Specific HDACs which play a major role need to be explored in subsequent studies. Overall, in addition to the direct hyperacetylation of autophagy genes promoters, the nuclear translocation of TFEB triggered by TSA may participate in TSA-induced autophagy.

### 2.4. TFEB Is Involved in TSA-Induced Autophagy and Lysosome Biosynthesis in Human Trophoblast Cells

**Figure 4:**
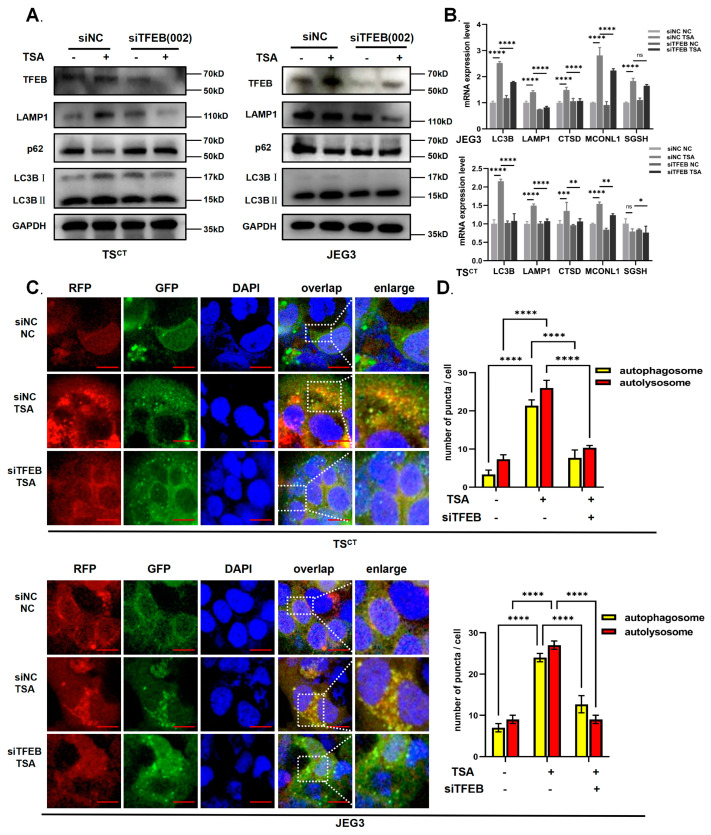
**TFEB was involved in TSA-induced autophagy in trophoblast cells.** (**A**) Western blot analysis of autophagy and lysosomal specific proteins LC3BII/I, p62, TFEB and LAMP1 in trophoblast cells with or without TFEB konockout for 48–72 h before TSA treatment (0.25 μmol/L, 12 h). (**B**) RT-qPCR analysis revealing the downstream target genes of TFEB including LAMP1, CTSD, MCONL1, SGSH, LC3B in TS^CT^ cells and JEG3 cells with or without TFEB konockout for 48–72 h before TSA treatment (0.25 μmol/L, 12 h). (**C**,**D**) Human TS^CT^ cells and JEG3 cells were transfected with Ad-mCherry-GFP-LC3B adenovirus with or without TFEB konockout and then treated with TSA. The numbers of red acidified puncta (GFP^−^mCherry^+^) versus neutral puncta (GFP^+^mCherry^+^) per cell were examined and quantified by the confocal microscopy. Scale bars = 20 μm. Statistical analyses were performed by Student’s *t*-test and One-way ANOVA; The data were presented as the mean ± SD. (* *p* < 0.05, ** *p* < 0.01, *** *p* < 0.001, **** *p* < 0.0001 and ns means non-significant). All experiments were performed in triplicate (*n* = 3).

To ascertain whether TFEB plays a significant role in TSA-induced autophagy and make the contribution more convincingly, we then performed gene silencing using siRNA, targeting TFEB. Of the three targets of siTFEB, TFEB siRNA-002 was the most effective for TFEB knockout, which was used in subsequent experiments along with control siRNA. As expected, the down-regulation of TFEB partially prevented the accumulation of the autophagy marker protein LC3B-II while it upregulated the expression of p62 compared with the TSA treatment alone (Figure 4A); the expression of autophagolysosomal-related proteins was also declined in mRNA levels compared to the control siRNA group (Figure 4B). Under the confocal microscope, we observed that the number of autolysosomes (GFP^-^mCherry^+^puncta) and autophagosomes (GFP^+^mCherry^+^puncta) all decreased after TFEB was down-regulated in Ad-mCherry-GFP-LC3B adenovirus transfected trophoblast cells. This suggested that autophagy was attenuated (Figure 4C,D), which was consistent with the results of Western blot analysis and RT-qPCR. Collectively, these results demonstrated that TFEB is involved in TSA-induced autophagy and lysosome biosynthesis, since TFEB deficiency partially prevented the TSA-induced increase in the expression of these genes.

### 2.5. HDAC Inhibitor TSA Interferes with Trophoblast Growth

**Figure 5:**
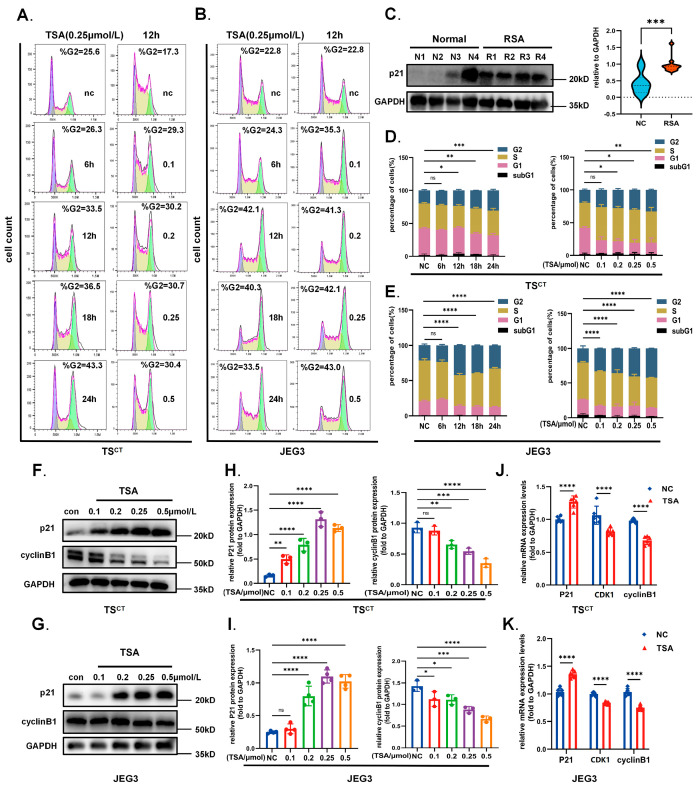
**TSA promoted G2 cycle arrest in trophoblast cells.** (**A**,**B**,**D**,**E**) The cell cycle phases of Human TS^CT^ cells and JEG3 cells after TSA treatment for different time and concentration were determined using flow cytometry assays. The percentages of cells in the G1, S, and G2/M phases are shown in the bar chart. (**C**) Western blot revealing the p21 expression in human first trimester villi from normal (*n* = 20) and RSA (*n* = 15) patients, quantified by densitometric analysis and normalized to GAPDH. (**F**–**I**) The G2 phase arrest associated proteins p21 and cyclinB1 were examined by Western blot analysis after treated with the different concentration of TSA for 12 h in TS^CT^ cells and JEG3 cells. Quantitative analysis were performed by ImageJ. (**J**,**K**) Relative mRNA expression of p21,CDK1 and cyclinB1 were estimated after TSA treatment (0.25 μmol/L, 12 h) using RT-qPCR. Statistical analyses were performed by Student’s *t*-test and One-way ANOVA; All the data were presented as the mean ± SD. (* *p* < 0.05, ** *p* < 0.01, *** *p* < 0.001, **** *p* < 0.0001 and ns means non-significant). All experiments were performed in triplicate (*n* = 3).

**Figure 6:**
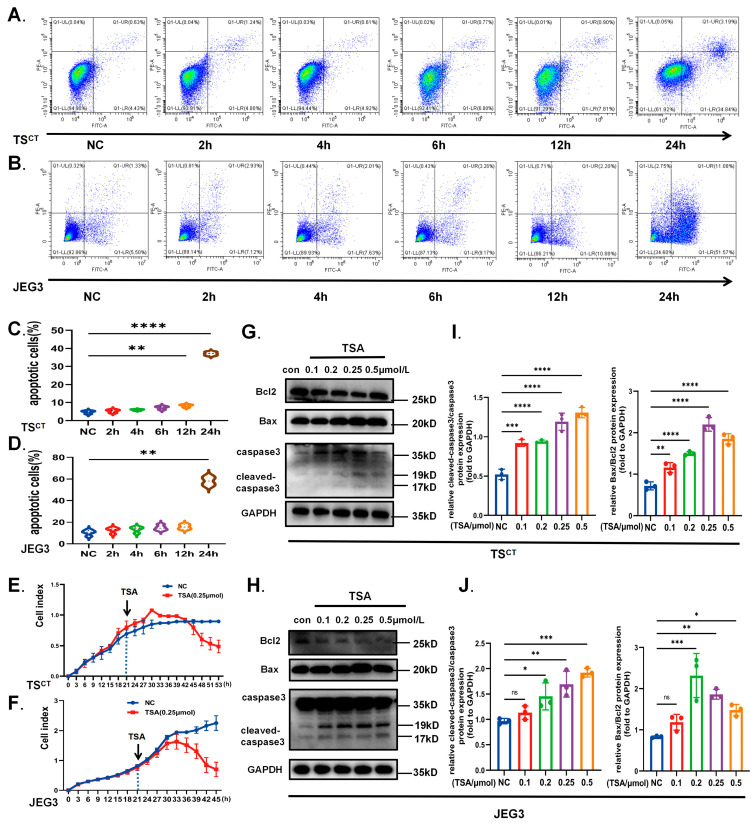
**TSA inhibited growth and promoted apoptosis of trophoblast cells.** (**A**,**B**) The apoptotic rates in different periods of Human TS^CT^ cells and JEG3 cells after TSA (0.25 μmol/L) treatment for different time were detected using flow cytometry assays. The percentages of apoptotic cell summation are shown in the (**C**,**D**). (**E**,**F**) The RTCA visually shows the growth curve of cells before and after treatment of TSA (0.25 μmol/L). (**G**–**J**) The apoptosis-associated proteins were examined by Western blot analysis after treated with the different concentration of TSA for 12 h in TS^CT^ cells and JEG3 cells. Quantitative analysis were performed by ImageJ. All the data were analyzed using One-way ANOVA and presented as the mean ± SD. (* *p* < 0.05, ** *p* < 0.01, *** *p* < 0.001, **** *p* < 0.0001 and ns means non-significant). All experiments were performed in triplicate (*n* = 3).

The steady growth of trophoblast cells during early placental development is crucial to maintain pregnancy, whose abnormity can initiate miscarriage [22]. Western blot analysis from our early gestational villus samples also confirmed that cell cycle arrest protein p21 increased in the RSA group compared with the control (Figure 5C). We then focused on the effect of TSA on trophoblast growth. Although the role of VPA is essential in the early culture of TS^CT^ cells, apoptosis and cycle arrest can still be observed with the increasing concentration of the additional treatment of TSA. Both TS^CT^ cells and JEG3 cells that treated with different concentration of TSA for 12 h or 0.25 μmol/L TSA for different time were all stably arrested in the G2 phase, as indicated by the flow cytometric analysis after PI staining (Figure 5A,B,D,E). Results of Western blot and RT-qPCR both revealed that the expression of p21 was markedly upregulated while the cyclinB1 and CDK1, which were mainly involved in the G2/M phase transition, declined after the treatment of TSA (Figure 5F–K). Meanwhile, the specific proapoptotic effect of high concentration TSA on trophoblast was confirmed. As expected, apoptosis-related markers including cleaved-caspase3 and Bax all increased in a dose-dependent manner after TSA treatment, while the anti-apoptotic protein Bcl2 decreased compared to the control (Figure 6G–J). Moreover, apoptosis analysis using Annexin-V/PI staining also confirmed that TSA increased the accumulation of early and late apoptotic cells, and the late apoptotic induction was more obvious. Notably, the dynamics changes of G2 stagnation and apoptosis induced by TSA were not obvious at the early stage. Cell cycle arrest mainly occurred after 6 h of TSA treatment, while apoptosis mainly happened after 12 h (Figure 6A–D). The RTCA real-time monitoring results manifested more intuitively that the trophoblast growth was inhibited, and the cell index decreased sharply after 10 h of TSA treatment (Figure 6E,F), suggesting that the occurrence of biological behavior change was later than the induction of autophagy by TSA. Therefore, we speculated that the TSA-induced autophagy may be related to the apoptosis and cycle arrest in trophoblast cells.

### 2.6. TFEB-Dependent Autophagy Contributes to the Growth Inhibition Effect of HDAC Inhibitor TSA on Trophoblast Cells

**Figure 7:**
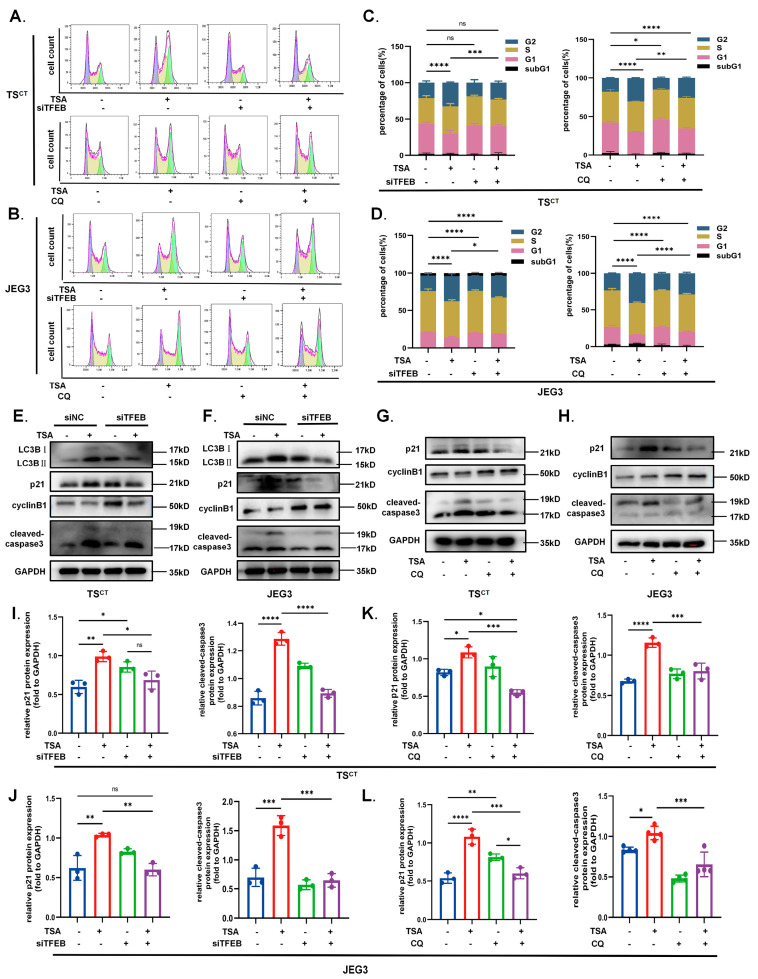
**Autophagy inhibition reversed the effect of the TSA on apoptosis and G2 cycle arrest in trophoblast cells.** (**A**,**B**) The Human TS^CT^ cells and JEG3 cells which pre-transfected with siNC and siTFEB for 48–72 h before TSA treatment (0.25 μmol/L, 12 h) or add an autophagy inhibitor (CQ 20 μmol/L) for last 6 h during TSA treatment were collected for cell cycle assay. (**C**,**D**) The percentages of cells in the G1, S, and G2/M phases are shown in the bar chart. (**E**,**F**) TS^CT^ cells and JEG3 cell transfected with siNC and siTFEB for 48–72 h before TSA treatment were collected for Western Blot analysis of LC3BII/I, p21, cyclinB1 and cleaved-caspase3. (**G**,**H**) TS^CT^ cells and JEG3 cell were treated with TSA for 12 h, with or without 20 μmol/L CQ for last 6 h before being collected for Western Blot analysis of proteins associated with apoptosis and G2 cycle arrest. (**I**–**L**) The bar chart is normalized relative expression of p21 and cleaved-caspase3 from three independent experiments, as indicated above. Statistical analyses were performed by Student’s *t*-test and One-way ANOVA; All the data were presented as the mean ± SD. (* *p* < 0.05, ** *p* < 0.01, *** *p* < 0.001, **** *p* < 0.0001 and ns means non-significant). All experiments were performed in triplicate (*n* = 3).

Whether overactivated autophagy functions as an independent phenotype remains unclear based on its context-dependent character [39]. To further identify whether TFEB-dependent autophagy participates in the growth inhibition induced by TSA and its potential function, we pre-treated with siTFEB transfection for 48–72 h and used the well-known autophagy inhibitor CQ during the last 6 h of TSA treatment to down-regulate autophagy levels. Then, changes in apoptosis and cycle arrest were evaluated again. As illustrated in Figure 7, we observed that TFEB knockdown partially alleviated the TSA-induced apoptosis and cycle arrest in both Human TS^CT^ cells and JEG3 cells. CQ treatment demonstrated similar effects. The flow analysis of a periodic graph revealed that the TSA-induced G2 arrest was attenuated to varying degrees after the down-regulation of autophagy while the TFEB knockout was more effective than CQ (Figure 7A–D). In addition, according to Western blot analysis, the TSA-induced p21 expression was significantly decreased in TFEB knockout or CQ-treated cells, while the cyclinB1 was slightly increased, further confirming the negative function of autophagy under hyperacetylation conditions (Figure 7E–L). Analogously, apoptosis demonstrated similar changes, and the autophagy blocking partially reversed the rise of the apoptotic marker cleaved-caspase3, suggesting that the cytotoxic effect of TSA seemed to be weaker (Figure 7E–L). All of the above results demonstrated that overactivated autophagy contributed to the trophoblast growth inhibition under hyperacetylation despite its essential and normal function as a conserved metabolic process. TFEB played an important role as one of the drivers in TSA-induced autophagy; an appropriate intervention in TFEB-mediated autophagy may alleviate hyperacetylation-induced damage to trophoblast cells.

### 2.7. Vivo Experiments: Autophagy Inhibition Partially Reverses the Effects of the TSA-Mediated Acetylation on Murine Abortion Rate during Early Pregnancy

**Figure 8:**
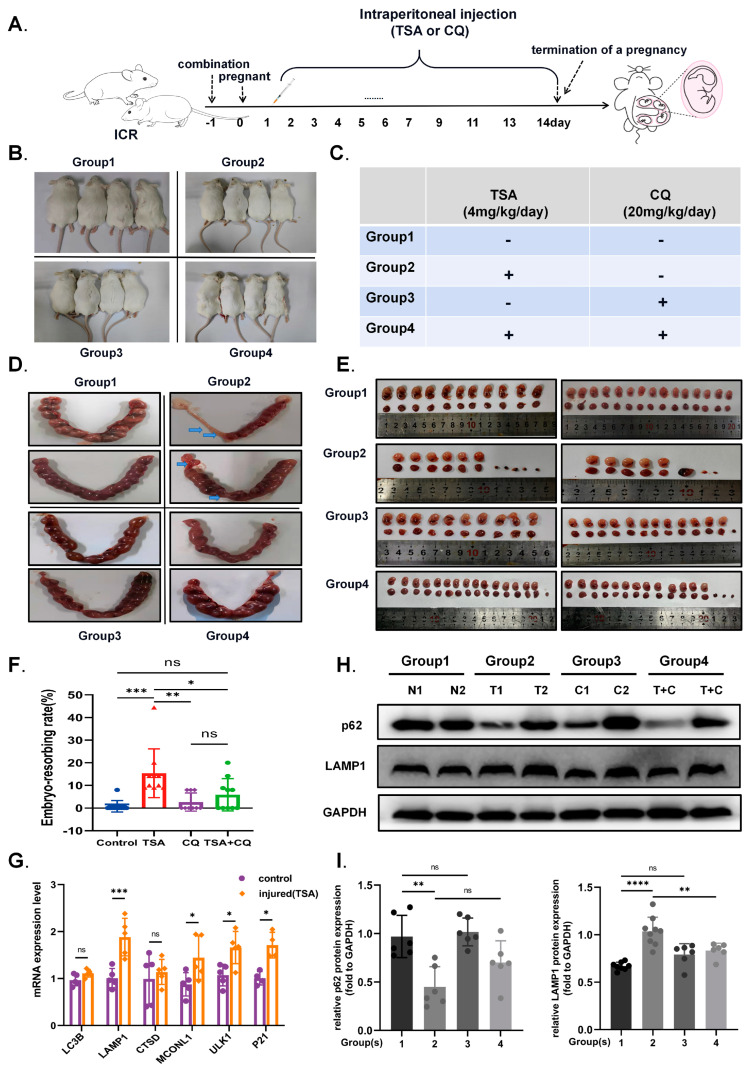
**Autophagy was involved in the effects of TSA on murine embryo-resorbing rate during early pregnancy.** (**A**) Schematic diagram of the animal experimental design. (**B**,**C**) Specific grouping of animal experiments and corresponding treatment. (**D**,**E**) Effects of TSA and CQ on the number of resorbed embryo in different groups. The blue arrows represent aborted placenta. (**F**) The statistics of embryo-resorbing rate after TSA alone and combined with autophagy inhibitors treatment of CQ. (**H**) The autophagy and lysosomal related proteins p62 and LAMP1 expressions of placental tissue in different groups. (**I**) Quantitative Western Blot analysis were performed by ImageJ. (**G**) Quantitative mRNA levels detection of autophagy related genes in placental tissue of control and TSA treated mice. Statistical analyses were performed by Student’s *t*-test and One-way ANOVA. All the Data were presented as the mean ± SD. (* *p* < 0.05, ** *p* < 0.01, *** *p* < 0.001, **** *p* < 0.0001 and ns means non-significant). All experiments were performed in triplicate (*n* = 3).

For in vivo experiments, we studied the physiological role of autophagy using ICR mice. The pregnant ICR mice after mating were divided into four groups, followed by treatment with TSA to create a hyperacetylation state with or without an autophagy inhibitor CQ to rescue. The experimental schematic diagram is presented in Figure 8A–C. Macroscopically, we calculated the embryo-resorbing rate (%) in different groups as the comparative index to evaluate the effect of TSA during early pregnancy in mice. In addition, the dynamic relationship between autophagy, HDAC expression and abortion rate was revealed by comparing the expression of related genes in placental tissue from normal pregnancy mice and aborted mice. As vividly shown in Figure 8D,E, TSA (4 mg/kg/day) treatment during early pregnancy for the first fourteen days induced the abortion of mice compared to the control group, while the autophagy inhibitor CQ alone had no significant effect. Furthermore, when the autophagy inhibitor CQ (20 mg/kg/day) were added along with TSA treatment to down-regulate autophagy levels, we observed that the elevated embryo-resorbing rate was partially reversed, although it still occurred (Figure 8F), which also provided evidence for the negative effect of excessive autophagy induced by hyperacetylation. Moreover, the expression of autophagy and the lysosomal specific marker protein p62 and LAMP1 (Figure 8H,I) increased simultaneously in the placental tissue of aborted mice. Upregulated mRNA levels of autophagy- lysosome-related genes were also observed in the placental tissue of mice, which was injected with TSA and miscarried (Figure 8G), indicating that TSA-mediated hyperacetylation and autophagy are associated with the miscarriage. These confirmed again that autophagy serves as a metabolic mechanism normally but plays a negative role in the hyperacetylated state of early pregnancy.

## 3. Discussion

In this research, we systematically investigated the effect of epigenetics and placental autophagy on RSA, observing that the down-regulation of histone deacetylases in the villi of RSA patients is usually accompanied with excessive autophagy. Our findings proposed for the first time that imbalanced acetylation levels can induce placental autophagy and interfere with trophoblast growth during early pregnancy, which may contribute toward the onset of RSA. In addition, we explored the mechanism of acetylation modification on placental autophagy at the transcription level and targeted the non-histone TFEB. Increased nuclear localization and transcriptional activity of TFEB were observed to be involved in TSA-induced excessive autophagy. Moreover, we used Human TS^CT^ cells as our powerful tool and validated that with the human trophoblast cell line JEG3, finding that overactivated autophagy has a negative influence on trophoblast growth under hyperacetylation despite its normal housekeeping roles, which is also supported by integrated data from abortion mice models and clinical RSA samples. TFEB, as one of the drivers in TSA-induced autophagy, played an important role in this process. Our research suggests that hyperacetylation and excessive autophagy in the villi during the first trimester may be one of the predictors for pregnancy outcome, and TFEB can become a potential target for therapy.

Epigenetic-related research has recently become a leading-edge field in life science. The accumulated evidence has established a strong link between epigenetic modification and early embryogenesis based on the long-term metabolic epigenome reprogramming induced by reproduction [40,41]. Protein acetylation modification is one of the major epigenetic mechanisms that widely exist during early pregnancy. As core regulators, HDACs have a profound effect on transcriptional activity and important cellular events [42,43]. The important endogenous pan-HDAC inhibitor butyrate was reported to affect pregnancy outcome via regulating progesterone synthesis [44]. Butyrate can deactivate and disinhibit the pyruvate dehydrogenase complex (PDC) to increase the conversion of pyruvate to acetyl-CoA, while acetyl-CoA upregulates the mitochondrial melatonergic pathway [45]. Melatonin is also a significant regulator of autophagy and mitophagy, with differential effects dependent upon wider cellular processes [46]. However, there are no data as to whether TSA does likewise, which is important to explore in future research. In addition, convincing studies have identified H3K27 acetylation as a key factor involved in the epigenetic regulation of gene expression during placental development, which, in turn might be related to impaired fetal growth [47]. H3K27 hyperacetylation is also characteristic of severe preeclampsia; cytotrophoblast from severe preeclampsia has been reported to have significantly increased acetylation of H3K27, which is normally down-regulated at term [18]. On the contrary, the down-regulation of acH3K9 has also been found in the syncytiotrophoblast and extra-villous trophoblast of GDM patients, indicating that dynamic changes of acetylation are closely associated with pregnancy disorders [48]. However, little is known about the role of exogenous HDACI-medicated acetylation during the pathological course of RSA. Here, we speculated on the correlation between the imbalance of HDAC expression and RSA by targeting trophoblast function, hoping to reveal the pathogenesis of unexplained RSA from the perspective of epigenetics. As expected, we found that the HDACI-mediated hyperacetylation disturbs the growth of trophoblast cells during early pregnancy, consistent with the generally down-regulated HDACs expression in the villi from RSA patients. Notably, excessive placental autophagy is always induced simultaneously, contributing to hyperacetylation-mediated abnormal pregnancy outcomes.

It is generally accepted that the establishment of proper pregnancy mainly depends on the biological behavior of the trophoblast. Strikingly, the histological and behavioral features of the trophoblast during placentation are similar to those of tumors [19,40,49]. The role of the aberrant epigenetic regulation in tumor development is well-known. Many lines of evidence emphasize that HDACI-mediated hyperacetylation can induce the autophagy of tumors, affecting the behavior of tumors and exerting an anticancer effect [16,40]. Autophagy, as an important physiological process, is regulated by a series of molecular mechanisms and is closely related to the function of trophoblast cells during early gestation. So far, the role of autophagy in pregnancy complications is thought to be two-sided. For example, there is controversy about the level of placental autophagy in women with preeclampsia, with several reports suggested that autophagy is hyperactivated in PE [50,51], while others proposed that the degradation pathway is impaired [52]. There have also been some studies about the correlation between RSA and autophagy, mainly focusing on immunoinflammatory factors. Epigenetic studies on the mechanism of autophagy regulation are limited. In our study, the human TS^CT^ cell was used as powerful research tool and the JEG3 cell was used for further validation. TSA, a broad-spectrum deacetylase inhibitor, can down-regulate HDAC expression and simulate the hyperacetylation level. We observed that autophagy signature proteins were stimulated, confirming the activation effect of HDACIs on placental autophagy in trophoblast cells, although the specific HDAC, which plays a major role, needs to be further explored in subsequent studies. Moreover, as the role of autophagy is environment-dependent, we explored whether it serves as a saving mechanism for survival or contributes to death under hyperacetylation conditions. The negative effects induced by TSA were alleviated after autophagy was down-regulated, indicating that autophagy may partially mediate the acetylation-caused damage of the trophoblast.

To further explore the molecular basis of the HDACI-induced autophagy, our research focused on transcriptional levels and identified a key non-histone TFEB based on characteristics of post-translational modification. TFEB is a master regulator of the autophagy-lysosome pathway [52,53]. A study has confirmed that the acetylation of TFEB can alleviate the pathological process of Alzheimer’s disease, a process independent of the MTORC1 pathway [32]. However, the role of TFEB in pregnancy has only been reported to be associated with PE, with a reduced expression and nuclear translocation in the placental trophoblast of PE patients [52]. Notably, here, we found for the first time that the expression of TFEB and its downstream gene LAMP1 is significantly increased in the trophoblast layer of RSA villi during early pregnancy. Immunohistochemistry verified the increased colocalization of TFEB with the nucleus, which is consistent with the overactivated autophagy in RSA. This conclusion has also been confirmed in vitro. TSA-promoted lysosome biogenesis and the nuclear translocation of TFEB in trophoblast cells and declined autophagy levels can be observed after TFEB knockout. Up to now, our findings have provided insights into the possible mechanism underlying the hyperacetylation-induced autophagy. We cannot exclude that the regulation of these autophagy genes is also linked to histone acetylation levels within their promoters. However, the results suggest that TFEB is at least partially involved in hyperacetylation-mediated autophagy during early pregnancy. Great importance to HDAC expression and autophagy levels should be taken, which might be predictors for pregnancy outcomes.

However, the following limitations need to be considered: Firstly, we investigated the overall level of HDAC activity under the treatment of broad-spectrum HDAC inhibitor TSA and found that the knockdown of HDAC1, 2, 5, 6 alone can also promote the nuclear translocation of TFEB in TS cells, but a further exploration of the specific HDAC subtypes that play a dominant role is needed to provide more clues about the relationship between HDACs, placental autophagy and pregnancy outcomes, which may provide more practical implications. Moreover, it is well known that MTOR-mediated phosphorylation is the most common post-translational modification affecting TFEB transcriptional activity, but whether TFEB activation and lysosomal function induced by TSA are independent of MTORC1 was not investigated in our study. In terms of mechanisms, a TFEB knockout mouse model will become the best choice to demonstrate that TFEB-dependent autophagy plays a role in inducing embryo resorption in the future. Additionally, we confirmed the negative role of autophagy in the hyperacetylation status, suggesting that acetylation and autophagy levels can be used as predictors for the pregnancy outcome. However, other intervention strategies to alleviate excessive autophagy should be investigated, especially targeting specific proteins in the autophagy pathway, for example, by improving the expression of HDACs to restore the homeostasis of acetylation and exploring the targeted inhibition of autophagy pathways such as TFEB. All of these may provide a new therapeutic target for RSA.

In summary, our study first makes a preliminary investigation on the regulation of placental autophagy from the perspective of epigenetics, proving that the maintenance of balanced acetylation levels in villi during the first trimester is important for trophoblast development, and hyperacetylation and its induced autophagy may contribute toward the onset of RSA. Notably, we suggest that monitoring villous autophagy and acetylation levels can help predict recurrent abortion in RSA patients. In terms of mechanism, we confirm that HDACI can not only promote histone acetylation but also induce autophagy at the transcriptional level by regulating non-histone TFEB and partially upregulating placental autophagy by increasing the nuclear translocation and transcriptional activity of TFEB. Additionally, we provide some evidence that excessive autophagy is involved in the growth inhibition of trophoblast, TFEB plays an important role in this process, providing novel potential therapeutic targets for further exploration and RSA treatment.

## 4. Materials and Methods

### 4.1. Tissue Collection and Preparation

Villus samples were obtained from individuals with a clinically normal pregnancy (*n* = 20) or recurrent spontaneous abortion (RSA) (*n* = 15). All participants came from the Sir Run Run Shaw Hospital, Zhejiang University Medical College and gave written informed consent. The design and conduct of this study were reviewed and approved by the Ethics Committee of Sir Run Shaw Hospital affiliated to the School of Medicine, Zhejiang University.

General information for the two groups is presented in Table 1. Detailed patient information can be found in the Supplementary Figure S3.

(1) **Normal pregnancy:** 20 healthy women who asked for legal termination of pregnancy (years of age range from 20 to 35; gestational ages range from 6 to 8 weeks). Each sample of gestational villus was gathered in sterile conditions and went through several washes in sterile pre-chilled PBS. Part of the tissue collected was fixed in 10% formalin solution for immunohistochemicals, while the other part was frozen in liquid nitrogen and stored at −80 °C for Western blot and RT-qPCR extraction.
(2) **Recurrent spontaneous abortion** (**RSA**)**:** All cases were unexplained recurrent spontaneous abortions but had normal karyotypes. They were comparable to normal pregnancy samples in terms of gestational age. The villus samples were taken within one week after the embryo was confirmed to be terminated. Confounding factors were excluded such as anatomical abnormalities, metabolic disorders or other autoimmune diseases.

### 4.2. Human Trophoblast Stem (TS) Cell Isolation and Cell Culture

Human TS^CT^ cell (extracted from first-trimester placental villi) and human-derived trophoblast cell line JEG3 were used in this experiment.

Human TS^CT^ cells were extracted from first-trimester placental villi of 3 healthy mothers. The detailed information is shown in Table 2.

The isolation and culture of TS cells were performed according to published protocols [3]. Briefly, the first-trimester placental villi were obtained in a sterile condition and placed on ice. After being dissected and minced into small pieces, they were washed in PBS and enzymatically digested three times with a mixture containing TrypLE and Accumax for 20 min at 37 °C. The EasySep phycoerythrin (PE)-positive selection kit was used to immunomagnetically purify the cytotrophoblasts (CTBs). The selected cells then were seeded into 6-well plates precoated with collagen IV, and sustained and cultured with TS media [6] (Supplementary Figure S4). CTB cells were passaged at about 90% confluency. We were able to obtain proliferative TS^CT^ cells from at least 1000 CTB cells. Cells at passages 10–30 were used for identification and differentiation assays. Cells were passaged using TrypLE incubated for 10 min at 37 °C, at about 90% confluency. TS^CT^ cells were further identified by testing the differentiation potential.

For ST differentiation, TS^CT^ cells were inoculated in Col IV pre-coated plates and cultured in ST medium (Supplementary Figure S4) for 2–3 days, then TS cells were fixed in 4% (*w*/*v*) paraformaldehyde for IF identification [4].

For EVT differentiation, TS^CT^ cells were inoculated in Col IV pre-coated plates and cultured in EVT medium (Supplementary Figure S4). Shortly after the cells were suspended in the medium, Matrigel with a final concentration of 2% (*v*/*v*) was added. The medium was then replaced with an NRG1-free EVT medium after 2 days, with a final concentration of 0.5% Matrigel added. The cells were transferred to a new Col IV-coated plate after 3 days of culture, using a EVT medium without NRG1 and KSR for another 2 days. Differentiated cells were collected on days 5 and 8 for identification [4].

Identification: CTB marker—CDH1 and TEAD4; ST marker—β-hCG; EVT marker—HLA-g. These were used for the identification of TS^CT^ (Supplementary Figure S4). 

The JEG3 cells were obtained from BNCC. Cells were cultured in 6 cm petri dishes using EMEM medium, supplemented with 10% Fetal Bovine Serum (FBS) and 1% Penicillin–Streptomycin Solution; cells were passaged using trypsin-EDTA incubated for 2–3 min at 37 °C.

All cells were free from contamination with bacteria, fungi or mycoplasma and passaged under aseptic conditions at about 90% confluency. Cultures were maintained in a humidified atmosphere with 5% CO_2_ at 37 °C.

### 4.3. Reagents and Antibodies

The important reagents used in this experiment are as follows: Trichostatin A (TSA, Selleck.cn, 58880-19-6, Houston, TX, USA); Chloroquine (CQ, C193834-50 mg, Aladdin, Shanghai, China); Ad-mCherry-GFP-LC3B (Beyotime, Shanghai, China, C3011). Tryple (Thermo Fisher Scientific, Waltham, MA, USA); collagen IV (354233, Corning, NY, USA); Accumax (Innovative Cell Tech, San Diego, CA, USA); DMEM/F12 (1:1) (11320-033, Thermo Fisher Scientific, USA); EasySep phycoerythrin (PE)-positive selection kit (Stemcell Technologies, Vancouver, BC, Canada); EMEM medium (Sigma, Cibolo, TX, USA); trypsin-EDTA; Penicillin-Streptomycin Solution (BL505A; Biosharp; Hefei, China); Fetal Bovine Serum (FBS) (Hyclone, Logan, UT, USA); PBS (GNM20012-, Genom, Haining, China) RNA-Quick Purification Kit (RN001, ES Science, Chengdu, China); HiScript II Reverse Transcriptase (R201-1, Vazyme, Nanjing, China); SYBR Green Supermix (D7260, Beyotime, Shanghai, China), SDS-PAGE (PG113 and PG114, Epizyme Biotech, Shanghai, China); paraformaldehyde (MA0192, Meilunbio, Dalian, China); Triton X-100 (T8787, Sigma-Aldrich, St. Louis, MO, USA); Lipofectamine™ 3000 Transfection Reagent (L3000015, Thermo Fisher Scientific, USA); (HRP)-conjugated secondary antibodies (GK500710, Gene Tech, Shanghai, China). Hematoxylin solution (ab220365, Abcam, Waltham, MA, USA); diaminobenzidine (DAB) (GK500710, Gene Tech, Shanghai, China); Chemiluminescent HRP Substrate (P90720, Millipore, Burlington, MA, USA); Cell Cycle and Apoptosis Analysis Kit (C1052, Beyotime, Shanghai, China); Dead Cell Apoptosis Kit (V13242, Invitrogen, Waltham, MA, USA); Lyso-Tracker Red fluorescent probe (C1046, Beyotime, Shanghai, China).

### 4.4. RNA Extraction and Real-Time Quantitative PCR

All cells for analysis were seeded in 24-well plates or 6-well plates to receive the corresponding treatments. The villus tissues were minced, weighed and ground thoroughly. Total RNA samples were extracted with the RNA-Quick Purification Kit according to the manufacturer’s protocol. Prepared RNA samples were reverse-transcribed into cDNA using HiScriptII Reverse Transcriptase under reaction condition: 25 °C for 10 min, 50 °C for 30 min and 85 °C for 5 min.; SYBR Green Supermix was used to perform the real-time quantitative PCR program, which includes 40 cycles of the following: 95 °C for 15 s, 58 °C for 30 s, and 72 °C for 30 s. The relative specific gene expression was standardized to the reference gene GAPDH and then compared with corresponding control samples. All experiments were performed in triplicate. The sequences of the corresponding RT-qPCR primers are precented in Table 3:

### 4.5. Western Blot Analysis

Tissue protein samples were extracted with a frozen RIPA lysis buffer containing 1% protease and phosphatase inhibitors, while cells were lysed using the loading buffer. All protein samples were separated by 12.5% sodium dodecyl sulfate-polyacrylamide gel electrophoresis (SDS-PAGE) after heating at 100 °C for 15 min and then transferred to PVDF membranes. The membranes were blocked for 1 h at room temperature with 5% dried skimmed milk, then incubated with primary antibodies at 4 °C overnight. The antibodies used in this experiment are as follows: p62 (ab109012; 1:1000; Abcam, USA); LC3B (ab192890; 1:1000; Abcam, USA); GAPDH (ab181602; 1:1000; Abcam, USA); AcH2BK5 (ab40886; 1:1000; Abcam, USA); AcH3K27 (8171S; 1:1000; CST, Peachtree City, GA, USA); TFEB (ab267351; 1:1000; Abcam, USA); LAMP1 (ab25630; 1:1000; Abcam, USA); P21 (2947S; 1:1000; CST, USA); CyclinB1 (12231S; 1:1000; CST, USA); Caspase3 (9662S; 1:1000; CST, USA); Cleaved-caspase3 (9661S; 1:1000; CST, USA); Bax (5023S; 1:1000; CST, USA); Bcl2 (ab32124; 1:1000; Abcam, USA). After washing three times in Tris-buffered saline with Tween 20 (TBST) buffer on the second day, the membranes were hatched with Horseradish peroxidase (HRP)-conjugated anti-rabbit (1:5000) or anti-mouse secondary antibodies (1:5000) for 1 h at room temperature. Subsequently, immunoreactions were captured by ChemiDoc Touch Imaging System (Bio-Rad, Hercules, CA, USA). ImageJ software (win64 version) was used to quantify the intensities of the Western blotting bands and normalized to DAPDH.

### 4.6. Immunofluorescence Staining

Cells (Human TS^CT^ cells /JEG3 cells) were used for immunofluorescence. Cells were seeded into confocal dishes, fixed with 4% paraformaldehyde for 30 min after a series of treatments and then permeabilized with 0.5% Triton X-100 for 15 min at room temperature. After blocking in 10% bovine serum albumin (BSA) diluted with PBS for 30 min, sections were incubated with primary antibodies overnight at 4 °C. The primary antibodies used in cells are as follows: TFEB (ab267351; 1:1000; Abcam, USA). After being rewarmed and washed with TBST buffer the next day, the cells were incubated with Alexa Fluor 488 or Alexa Fluor 568 conjugated goat secondary antibody (1:500) for another one hour. Images and fluorescence intensity were caught by confocal microscope (Zeiss LSM800, Jena, Germany) and representative cells were selected and photographed. A quantification analysis was performed using GraphPad Prism 9.0 version.

### 4.7. Immunohistochemistry of Paraffin Sections

Paraffin sections were deparaffinized and hydrated in xylene (1/2) and ethanol (100%-95%-75%) orderly for 40 min. Subsequently, they were boiled at 100 °C in citrate sodium buffer (pH 6.0) for 20 min to retrieve antigens. After endogenous peroxidase activity was suppressed by 3% H_2_O_2_, slides were blocked with 1% bovine serum albumin (BSA) for 1 h at room temperature and incubated with primary antibodies overnight at 4 °C. The primary antibodies used in cells are as follows: TFEB (ab267351; 1:1000; Abcam, USA); P62 (ab109012; 1:1000; Abcam, USA); rewarming at room temperature and washing with PBS the next day. Sections were incubated with horseradish peroxidase (HRP)-conjugated secondary antibodies for 1 h, which were tested by diaminobenzidine for next 5 min. A hematoxylin solution was used to stain nuclei and then the sections were sealed with gum after dehydration. Images were captured with an Axio Scope A1 (Zeiss, Jena, Germany).

### 4.8. RNA Interference (Transient Transfection of siRNAs) 

To down-regulate endogenous TFEB in Human TS^CT^ and JEG3 cells, three independent silencer-select siRNAs targeting the coding region of TFEB and negative control siRNA (siNC) were transfected using lipofectamine™ 3000 Transfection Reagent (L3000015, Thermo Fisher Scientific, USA). The reagent was applied according to the manufacturer’s instructions after the cells were cultured in 12-well plates and at a 60% cell confluence. The transfection time was 48–72 h. TFEB 002 was selected for further experimentation based on the optimum efficiency of transfection confirmed by Western blot analysis and quantitative real-time PCR. The siRNAs were purchased from Ribo Biological Technology Co., Ltd. (Guangzhou, China). The targeting sequences of human TFEB was GAAAGACAATCACAACTTA.

### 4.9. Autophagy Adenovirus Transfection

Adenovirus vectors encodingLC3 (Ad-mCherry-GFP-LC3B) was purchased from (Beyotime, C3011, China) and used to quantify autophagic flow intuitively. Human TS^CT^ cells and JEG3 cells were seeded into 6-well plates and cultured for 24 h to reach about 60% confluency. Adenovirus was diluted with antibiotic-free medium according to the manufacturer’s instructions, with 1 mL added to each plate. After 4 h of culture, another 1 mL media was added and incubated for 20 h. The infected cells were passaged to confocal dishes to receive treatment accordingly and counterstained with DAPI for 10 min. The fluorescence intensity was detected by a confocal laser scanning microscope (Zeiss, LSM800, Jena, Germany). Images were analyzed by ImageJ software through counting the yellow vesicles (GFP^+^mCherry^+^puncta, representing autophagosomes) and red vesicles (GFP^-^mCherry^+^puncta, representing autolysosomes) in individual cells.

### 4.10. Examination of Lysosomal Acidification

The lysosomal acidification was detected by Lyso-Tracker Red according to the manufacturer’s instructions. Briefly, cells were inoculated into confocal dishes after treatments and then lysosome staining was performed. The reagent was diluted at 1:20,000 and added to confocal dishes for 30 min. The fluorescence intensity was captured by a confocal laser scanning microscope (Zeiss, LSM800, Jena, Germany) after several washes of cells. Lysosomes in the image were quantified by ImageJ.

### 4.11. Electron Microscopy

Cells were collected in clumps by digestion, centrifuged after the TSA treatment and fixed with 2.5% glutaraldehyde for 24 h. Then, they were postfixated with moderate 1% osmium tetroxide for 1 h, followed by 100 µL 2% uranium acetate staining for 30 min, then dehydrated dried with gradient ethanol (50%, 70% and 90%, respectively). They were rinsed with PBS 2–3 times between each step. The embedding agent and acetone were mixed in a ratio of 1:1 at room temperature for 2 h to permeate it. The mass of cells was cut into thin sections (150 nm) and analyzed using a Tecnai T10100 kV electron microscope (Philips, Amsterdam, The Netherlands) [9]. Samples were prepared and processed with the assistance of experts from the Center of Cryo-Electron Microscopy of Zhejiang University. Autophagic vacuoles were captured per 8300X field.

### 4.12. Nucleoplasmic Separation

Human TS^CT^ cells/JEG3 cells were treated with different concentration of TSA for 12 h. The nuclear and cytoplasmic extracts were started using nuclear extraction reagents (CyNIB) and cytoplasmic extraction reagents (CIB), according to the manufacturer’s instructions.

### 4.13. Apoptosis and Necrosis Analysis

After being treated with the indicated concentration of drugs (TSA and/or CQ) for a specific time or RNA interference, cells were harvested gently using trypsin-EDTA, centrifuged at 1200 rpm for 5 min and washed with PBS three times. Then, they were stained with FITC-conjugated Annexin V and propidium iodide (PI) from Invitrogen Cell Apoptosis Kit, as requested. Briefly, 5 μL FITC Annexin V and 1 μL 100 μg/mL PI solution was added, and the cells were vortexed gently and incubated for 15 min at room temperature in the dark. Finally, 400 μL 1× Annexin V binding buffer was added to each tube to stop the reaction. Apoptotic cells were measured using the flow cytometer. Images were analyzed by FlowJo software (10.8.1 version).

### 4.14. Cell Cycle Detection

Cells were seeded into 6-well plates and cultured for 24 h. Then, they were treated with TSA (0.25 μmol/mL) or transfected siRNA. According to the manufacturer’s instructions, cells (5 × 10^5^) were washed by PBS and fixed with 70% cold ethanol at 4 °C overnight. After washing twice with cold PBS, cells were stained with 500 μL mixture containing 25 μL PI solution and 10 μL RNase for 30 min at room temperature. The fluorescence intensities of viable cells were measured using the flow cytometer. Images were analyzed by FlowJo software.

### 4.15. Mice

Eight-week-old female and male ICR mice were purchased from Beijing SPF Biotechnology Corporation. All mice were healthy, not pregnant, randomly purchased, had no organic lesions and were not pretreated. All animals used were housed and bred in the Animal Facility of Sir Run Run Shaw Hospital, Zhejiang University under a standardized environment with a 12 h light/dark schedule and 20–24 °C temperature, in accordance with the institutional guidelines for laboratory animals. The animal treatment protocol was approved by the Zhejiang University Institutional Animal Care and Use Committee.

### 4.16. Mouse Experiments

Eight-week-old female and 10-week-old male ICR mice (weight: 20–30 g) were divided into four equal groups: (1) control group (saline intraperitoneal injection); (2) TSA intraperitoneal injection group (TSA: 4 mg/kg/day); (3) CQ intraperitoneal injection group (CQ: 20 mg/kg/day); (4) TSA + CQ intraperitoneal injection group (TSA: 4 mg/kg/day + CQ: 20 mg/kg/day). The day the vaginal plug appeared was defined as day 0.5 of gestation. The intraperitoneal injection was performed during the first 14 days. Pregnant mice were forced to terminate their pregnancies at the end of 14 days to analyze embryo absorption rates and implantation numbers. Placental tissues from mice in different groups during early pregnancy were collected for subsequent protein and RNA extraction. The percentage of fetal loss (embryo absorption rate) was figured as follows: percentage fetal loss (%) = A/(A + B) × 100 (A = the number of absorbed placenta or fetal loss sites; B = the number of surviving fetuses). 

### 4.17. Statistical Analysis

All of the statistical analyses and drawing of diagrams were performed by GraphPad Prism 9.0 version software. All experiments were repeated at least three times, and results were presented as the mean ± SD. Comparisons between two groups were performed using the unpaired Student’s *t*-test while One-way ANOVA was used for multiple analyses. The variance is similar between the groups that were statistically compared, and discrepancies were considered statistically significant if the *p* value was less than 0.05. The significance is demonstrated as follows: * *p* < 0.05; ** *p* < 0.01; *** *p* < 0.001; **** *p* < 0.0001.

## Figures and Tables

**Table 1:** Clinical sample.

Parameters	Normal (*n* = 20)	RSA *(n* = 15)	*p* Value (Normal vs. RSA)
Sample size (years)	20	15	-
Maternal age (years)	29.85 ± 5.17	31.33 ± 4.7	0.2421 (ns)
Gestational age (days)	50.15 ± 6.15	47.2 ± 4.44	0.1358 (ns)

ns means non-significant in maternal age and gestational age between normal pregnancy and recurrent spontaneous abortion.

**Table 2:** Summary of TS^CT^ Cell Derivation:

TS Line	Maternal Age (Years)	Gestational Age at D&C	Karyotype
TS^CT^ #1	31	8 weeks	46, XY
TS^CT^ #2	24	6 weeks	46, XX
TS^CT^ #3	32	7 weeks	46, XX

**Table 3:** The sequences of the corresponding RT-qPCR primers.

	Forward Primer	Reverse Primer
HDAC1	CTACTACGACGGGGATGTTGG	GAGTCATGCGGATTCGGTGAG
HDAC2	ATGGCGTACAGTCAAGGAGG	TGCGGATTCTATGAGGCTTC
HDAC3	CCTGGCATTGACCCATAGCC	CTCTTGGTGAAGCCTTGCATA
HDAC4	GGCCCACCGGAATCTGAAC	GAACTCTGGTCAAGGGAACTG
HDAC5	TCTTGTCGAAGTCAAAGGAGC	GAGGGGAACTCTGGTCCAAAG
HDAC6	AAGAAGACCTAATCGTGGGACT	GCTGTGAACCAACATCAGCTC
HDAC7	GGCGGCCCTAGAAAGAACAG	CTTGGGCTTATAGCGCAGCTT
HDAC8	TCGCTGGTCCCGGTTTATATC	TACTGGCCCGTTTGGGGAT
HDAC9	AGTAGAGAGGCATCGCAGAGA	GGAGTGTCTTTCGTTGCTGAT
HDAC10	CAGTTCGACGCCATCTACTTC	CAAGCCCATTTTGCACAGCTC
LAMP1	TCTCAGTGAACTACGACACCA	AGTGTATGTCCTCTTCCAAAAGC
CTSD	TGCTCAAGAACTACATGGACGC	CGAAGACGACTGTGAAGCACT
MAP1LC3B	GATGTCCGACTTATTCGAGAGC	TTGAGCTGTAAGCGCCTTCTA
BECLIN1	CCATGCAGGTGAGCTTCGT	GAATCTGCGAGAGACACCATC
ATG5	AAAGATGTGCTTCGAGATGTGT	CACTTTGTCAGTTACCAACGTCA
ATG7	CAGTTTGCCCCTTTTAGTAGTGC	CCAGCCGATACTCGTTCAGC
ATG12	CTGCTGGCGACACCAAGAAA	CGTGTTCGCTCTACTGCCC
MCOLN1	TTCGCCGTCGTCTCAAATACT	CTCTTCCCGGAATGTCACAGC
SGSH	ACGGAGGCTTTGAGAGTGG	GCATTGCGAAAGAGGAGGCT
P21	GATTCTGCCATACAAGGCTACAA	TGCCCTGGGTATAACTCCCAA
cyclinB1	AATAAGGCGAAGATCAACATGGC	TTTGTTACCAATGTCCCCAAGAG
CDK1	AAACTACAGGTCAAGTGGTAGCC	TCCTGCATAAGCACATCCTGA

## Data Availability

The original contributions presented in the study are all included in the article. Further inquiries can be directed to the corresponding authors. The study includes no data deposited in external repositories.

## Supplementary Materials

The supporting information can be downloaded at: https://www.mdpi.com/article/10.3390/ijms241511899/s1.
