# Supplementary material for: Dysregulation of Histone Deacetylases Inhibits Trophoblast Growth during Early Placental Development Partially through TFEB-Dependent Autophagy-Lysosomal Pathway
Source: Int J Mol Sci. 2023 Jul 25;24(15):11899. doi: 10.3390/ijms241511899 (PMC10418899; doi:10.3390/ijms241511899)

	Component	Concentration	Identifier and Company
TS medium	2-Mercaptoethanol	0.1 mM	Cat#21985023 Thermo Fisher Scientific, USA
	FBS	0.20% (v/v)	Cat#16141-079 Thermo Fisher Scientific, USA
	Penicillin-Streptomycin	1% (v/v)	Cat#15140122 Thermo Fisher Scientific, USA
	BSA	0.30% (w/v)	Cat#017-22231 Wako, Japan
	ITS-X supplement	1% (v/v)	Cat#094-06761 Wako, Japan
	L-ascorbic acid	1.5 µg/ml	Cat#013-12061 Wako, Japan
	EGF	50 ng/ml	Cat#053-07871 Wako, Japan
	CHIR99021	2 µM	Cat#038-23101 Wako, Japan
	A83-01	0.5 µM	Cat#035-24113 Wako, Japan
	SB431542	1 µM	Cat#031-24291 Wako, Japan
	VPA	0.8 mM	Cat#227-01071 Wako, Japan
	Y27632	5 µM	Cat#257-00511 Wako, Japan
ST(2D) medium	2-Mercaptoethanol	0.1 mM	Cat#21985023 Thermo Fisher Scientific, USA
	Penicillin-Streptomycin	0.5% (v/v)	Cat#15140122 Thermo Fisher Scientific, USA
	BSA	0.3% (w/v)	Cat#017-22231 Wako, Japan
	ITS-X supplement	1% (v/v)	Cat#094-06761 Wako, Japan
	Y27632	2.5 µM	Cat#257-00511 Wako, Japan
	Forskolin	2 µM	Cat#067-02191 Wako, Japan
	KnockOut Serum Replacement	4% (v/v)	Cat#10828028 Thermo Fisher Scientific, USA
ST(3D) medium	2-Mercaptoethanol	0.1 mM	Cat#21985023 Thermo Fisher Scientific, USA
	Penicillin-Streptomycin	0.5% (v/v)	Cat#15140122 Thermo Fisher Scientific, USA
	BSA	0.3% (w/v)	Cat#017-22231 Wako, Japan

	ITS-X supplement	1% (v/v)	Cat#094-06761<br>Wako, Japan
	Y27632	2.5 µM	Cat#257-00511<br>Wako, Japan
	Forskolin	2 µM	Cat#067-02191<br>Wako, Japan
	KnockOut Serum Replacement	4% (v/v)	Cat#10828028<br>Thermo Fisher Scientific, USA
	EGF	50 ng/ml	Cat#053-07871<br>Wako, Japan
EVT medium	2-Mercaptoethanol	0.1 mM	Cat#21985023<br>Thermo Fisher Scientific, USA
	Penicillin-Streptomycin	0.5% (v/v)	Cat#15140122<br>Thermo Fisher Scientific, USA
	BSA	0.3% (w/v)	Cat#017-22231<br>Wako, Japan
	ITS-X supplement	1% (v/v)	Cat#094-06761<br>Wako, Japan
	Y27632	2.5 µM	Cat#257-00511<br>Wako, Japan
	KnockOut Serum Replacement	4% (v/v)	Cat#10828028<br>Thermo Fisher Scientific, USA
	NRG1	100 ng/ml	Cat#5218SC<br>Cell Signaling Technology, USA
	A83-01	7.5 µM	Cat#035-24113<br>Wako, Japan

TS^{CT}

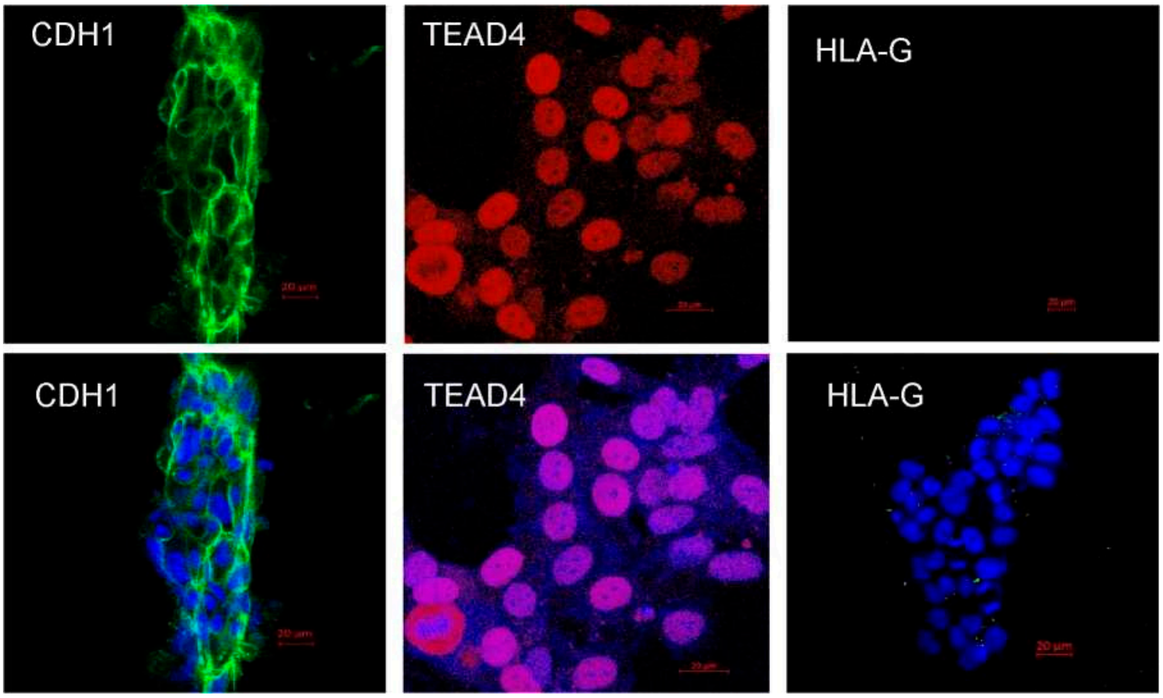

EVT-TS^{CT}

ST(2D) -TS_{CT}

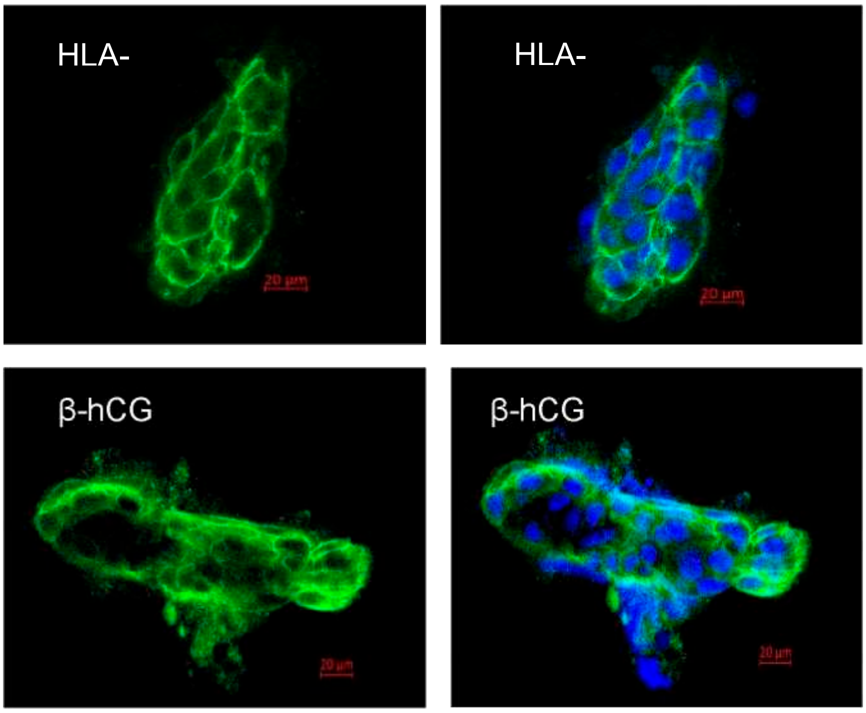

Supplement: Supplementary file 1 [file ijms-24-11899-s001.zip › identification and culture.pdf]
