# Supplementary material for: Dysregulation of Histone Deacetylases Inhibits Trophoblast Growth during Early Placental Development Partially through TFEB-Dependent Autophagy-Lysosomal Pathway
Source: Int J Mol Sci. 2023 Jul 25;24(15):11899. doi: 10.3390/ijms241511899 (PMC10418899; doi:10.3390/ijms241511899)
Supplement: Supplementary file 1 [file ijms-24-11899-s001.zip › Supplement figures.pdf]

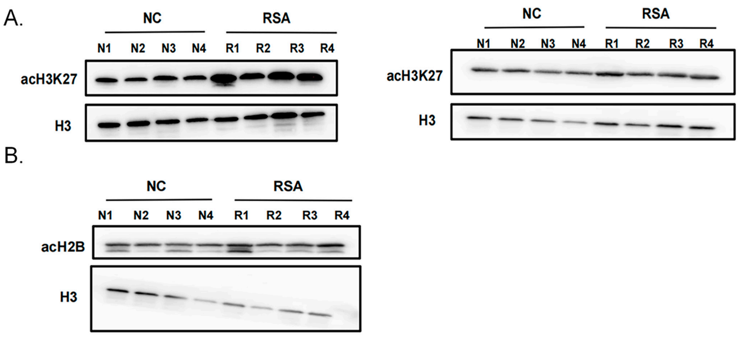

Supplementary Figure S1:
A,B. Human first trimester villus samples from normal (n=20)and RSA (n=15) patients were used for Western Blot analysis of acH2B and acH3. They were all normalized to H3.

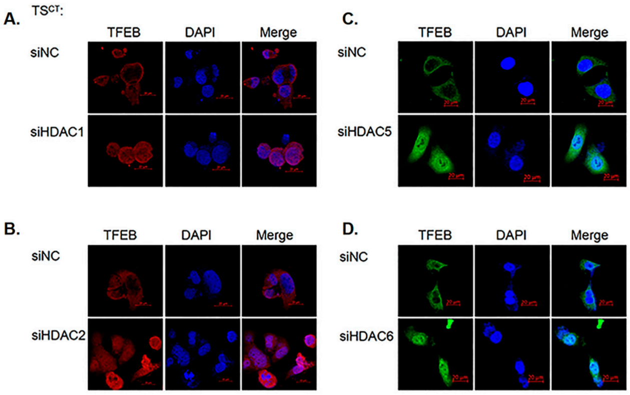

A,B,C,DImmunofluorescence detection the TFEB nucleus translocation in trophoblast cells with or without siHDAC1/2/5/6 for 48-72h. Scale bars=20μm.
